# Virtual Reality–Guided Meditation for Chronic Pain in Patients With Cancer: Exploratory Analysis of Electroencephalograph Activity

**DOI:** 10.2196/26332

**Published:** 2021-06-24

**Authors:** Henry Fu, Bernie Garrett, Gordon Tao, Elliott Cordingley, Zahra Ofoghi, Tarnia Taverner, Crystal Sun, Teresa Cheung

**Affiliations:** 1 School of Engineering Science Simon Fraser University Burnaby, BC Canada; 2 School of Nursing University of British Columbia Vancouver, BC Canada; 3 School of Rehabilitation Sciences University of British Columbia Vancouver, BC Canada; 4 School of Interactive Arts and Technology Simon Fraser University Surrey, BC Canada; 5 Surrey Memorial Hospital Fraser Health Authority Surrey, BC Canada; 6 Behavioural and Cognitive Neuroscience Institute Simon Fraser University Burnaby, BC Canada

**Keywords:** virtual reality, guided meditation, neurophysiology, electroencephalograph, EEG

## Abstract

**Background:**

Mindfulness-based stress reduction has demonstrated some efficacy for chronic pain management. More recently, virtual reality (VR)–guided meditation has been used to assist mindfulness-based stress reduction. Although studies have also found electroencephalograph (EEG) changes in the brain during mindfulness meditation practices, such changes have not been demonstrated during VR-guided meditation.

**Objective:**

This exploratory study is designed to explore the potential for recording and analyzing EEG during VR experiences in terms of the power of EEG waveforms, topographic mapping, and coherence. We examine how these measures changed during a VR-guided meditation experience in participants with cancer-related chronic pain.

**Methods:**

A total of 10 adult patients with chronic cancer pain underwent a VR-guided meditation experience while EEG signals were recorded during the session using a BioSemi ActiveTwo system (64 channels, standard 10-20 configuration). The EEG recording session consisted of an 8-minute resting condition (pre), a 30-minute sequence of 3 VR-guided meditation conditions (med), and a final rest condition (post). Power spectral density (PSD) was compared between each condition using a cluster-based permutation test and across conditions using multivariate analysis of variance. A topographic analysis, including coherence exploration, was performed. In addition, an exploratory repeated measures correlation was used to examine possible associations between pain scores and EEG signal power.

**Results:**

The predominant pattern was for increased β and γ bandwidth power in the meditation condition (*P*<.025), compared with both the baseline and postexperience conditions. Increased power in the δ bandwidth was evident, although not statistically significant. The pre versus post comparison also showed changes in the θ and α bands (*P*=.02) located around the frontal, central, and parietal cortices. Across conditions, multivariate analysis of variance tests identified 4 clusters with significant (*P*<.05) PSD differences in the δ, θ, β, and γ bands located around the frontal, central, and parietal cortices. Topographically, 5 peak channels were identified: AF7, FP2, FC1, CP5, and P5, and verified the changes in power in the different brain regions. Coherence changes were observed primarily between the frontal, parietal, and occipital regions in the θ, α, and γ bands (*P*<.0025). No significant associations were observed between pain scores and EEG PSD.

**Conclusions:**

This study demonstrates the feasibility of EEG recording in exploring neurophysiological changes in brain activity during VR-guided meditation and its effect on pain reduction. These findings suggest that distinct altered neurophysiological brain signals are detectable during VR-guided meditation. However, these changes were not necessarily associated with pain. These exploratory findings may guide further studies to investigate the highlighted regions and EEG bands with respect to VR-guided meditation.

**Trial Registration:**

ClinicalTrials.gov NCT00102401; http://clinicaltrials.gov/ct2/show/NCT00102401

## Introduction

### Background

Chronic pain (CP) is a common condition occurring in 1 in 5 Canadians [1], and it has limited effective treatment approaches. Mindfulness-based stress reduction (MBSR) has shown some evidence of efficacy in this area [2,3] and has also been used to treat other clinical conditions, such as migraine, depression, addiction, and substance misuse [2,4].

Mindfulness meditation encompasses a range of mental exercises that share a common focus on regulating attention and awareness to improve well-being [2-4]. It is described as the quality of being completely engaged in the present moment, free from distractions and judgments, and being aware of bodily sensations, thoughts, and feelings without getting caught up by them, and it is used as a therapeutic technique in MBSR [5,6]. These practices involve mental training that allows practitioners to develop their minds in specific ways to help them deal with stress and anxiety [7,8]. Although the clinical benefits remain somewhat controversial, it is generally viewed as a beneficial practice for mental well-being, stress reduction, and pain management [9-11].

A recent trend in MBSR practice has been the use of immersive virtual reality (VR) to help participants focus on meditative exercise [12-16]. However, to date, neurological studies have not been performed with VR-guided meditation practices. Therefore, this exploratory study sought to identify any identifiable neurological effects of VR-guided meditation practices using electroencephalographs (EEGs).

### Neurological Mindfulness Studies

Subjective reports of the benefits of mindfulness meditation have prompted investigations into the potential corresponding neurophysiological states. Exploration of fluctuations in brain wave voltage amplitude (power) topography and coherence (associated areas of activity) using EEG variations in neural activity assessed with functional magnetic resonance imaging and cortical evoked responses to visual and auditory stimuli that reflect the impact of meditation on attention [4,17-26]. However, findings remain speculative. EEG studies have previously reported modulation in α, θ, and γ band frequencies, generally with increased power and coherence during mediation [4,7,17,27]. Some studies theoretically conjectured how meditative states relate to EEG bandwidths. For example, Travis and Shear [28] suggested that focused attention (sustained attention is focused on a given object) increases γ band power, open monitoring (nonreactive monitoring of an ongoing experience) increases θ, and an automatic-self-transcending stage (transcending the procedures of the meditation) increases α [28]. Nevertheless, a consensus on what we currently know about how EEG forms correlate with meditation or how this may map onto stages of mental development or specific cognitive skills is yet to be reached [7,8].

The α frequency band lies between 8 and 12 Hz and is predominantly located in the occipital cortex. α waves are present in deep relaxation and sleep, usually when the eyes are closed. θ waves are characterized by oscillations in the 4-8 Hz band found in both cortical and subcortical structures. Increases in θ have been described during a variety of learning and recognition tasks, light sleep (including the rapid-eye-movement dream state), and deep meditation. θ and α power changes have been reported to increase in a number of meditation studies [4,17,19,29-31]. Moreover, γ is a higher frequency range generally regarded as between 30 and 50 Hz, although the range reported has varied substantially between 20 and 200 Hz across different studies [30]. This initial research suggests that γ is associated with a number of sensory and cognitive high-level information processing functions, such as semantic insights, learning, and neural plasticity. Peak γ frequencies around 40 Hz in bilateral hemispheres have only been observed in highly practiced meditators [7,30]. In addition, a posterior increase in γ activity may be related to enhanced perceptual clarity reported in some open monitoring (focusing on awareness itself) meditative processes [7,17,30].

### VR-Assisted Mindfulness

VR is rapidly developing as a new form of media and uses computer-generated audio-visual displays and hand controller user interfaces to produce a sense of immersion in a digital 3D environment. Instead of watching an image on a typical computer or video display, VR technologies provide an increased sense of presence by engaging the senses (sight, sound, and touch) in real-time stereoscopic audio-visual media where users can move around and explore the environment as if they were there.

### VR in Pain Management

One of the most common health care applications of VR is its use in pain management. Several studies have explored the value of MBSR in CP management, although the reported effect sizes for this technique have been typically mild to moderate, and regular adherence with meditative practice is problematic [3,32,33]. It has been suggested that combining mindfulness meditation within a VR intervention may help support acceptance and adherence to practice while having a synergistic effect on pain reduction through immersive VR distraction [34-36]. This remains an active area of research, as adjunctive VR strategies have been used successfully in the treatment of acute pain [37-41] and more recently have also been explored with CP [12,35,42,43]. The theoretical rationale behind why VR may enhance mindfulness skills is that VR provides the user with cognitive displacement by actively engaging in a coping activity that provides a profound sense of through presence in another world. Cognitive distraction is a common strategy in pain control and relies on creating competition for cognitive resources, that is, attention to a novel spatial orientation, and engaging within it reduces the perception of pain [32,33,44]. Therefore, immersive VR interventions using stereoscopic head-mounted displays (HMDs) have been proposed as powerful tools for providing visual, audio, cognitive, and emotional engagement [45-47]. In MBSR, VR experiences are typically accomplished using computer-simulated environments, stereoscopic headsets, and motion tracking to support a more immersive meditative experience. This was the approach taken in this study, and an EEG analysis was selected as a practical technique to assess neurophysiological activity while experiencing VR.

### Objectives

Overall, this inductive exploratory study sought to assess how EEG power of waveforms, their topographic mapping, and coherence measures altered in 3 main states during a VR-guided meditation experience in patients with cancer-related CP: at baseline (pre), during VR-guided meditation (med), and after VR-guided meditation (post). Moreover, we explored whether their pain level was associated with waveform power measures. We were particularly interested in the power, topography, and coherence of α, γ, and θ wave activity, and possible synchrony with VR activity, as other researchers have reported changes in these 3 waveforms with MBSR activities. Therefore, we were interested in determining whether prior findings were consistent with those observed during a VR experience. Specifically, the questions we sought to address in this study were as follows:

1. Were there any observable or significant changes between pre and during VR-guided meditation experiences and between pre and post VR-guided meditation experiences with the power, topographic changes, or EEG coherence in specific waveforms?
2. Was there any evidence that changes in the pain experienced by participants during a VR-guided meditation activity were associated with observable EEG changes?

## Methods

### Approach

An exploratory, single-subject design study was undertaken to compare EEG activity and pain levels before, during, and after VR-based meditation practice. This study was reviewed and approved by the University of British Columbia Clinical Research Ethics Board.

### Recruitment

A convenience sample of 10 participants was used and recruited from those in an existing randomized controlled trial (RCT; ClinicalTrials.gov: NCT 02995434) where patients with cancer were using VR as an adjunctive therapy to help manage their CP (Textbox 1). These participants were completing or had completed cancer treatment and experienced a range of cancer-related pain, including neuropathy, fibromyalgia, postsurgical pain, or an exacerbation of pre-existing pain.

**Randomized controlled trial eligibility criteria.**

**Eligibility Criteria**

- Aged ≥16 years, with a past or current diagnosis of cancer
- Prior or ongoing cancer treatment
- A chronic pain diagnosis (ongoing daily pain for ≥3 months, with a Neuropathic Pain Rating Scale of ≥4)
- Able to understand English (read and write)
- Normal stereoscopic vision
- Able to move their head up, down, left, and right and able to wear a virtual reality head-mounted display
- Sufficient fine motor control in one hand to use a game controller
- Have space at home for a computer and monitor

Participants were purposively recruited, focusing on recruiting those from the RCT cohort who had previously responded well to a VR-based meditation experience, with a self-reported reduction of a Visual Analog Scale for Pain ≥1. They were invited to participate in a single 2-hour VR-guided meditation experience, with EEG recorded in their home or at the university, and were offered a Can $100 (US $83) honorarium and expenses for taking part. As an exploratory study designed to establish methods and feasibility using a limited convenience sample, with an unknown effect size, the power to inform the sample size was not calculated a priori. This is acceptable for this type of inductive study [48].

### Equipment

EEG signals were recorded during the session using a BioSemi ActiveTwo system (BioSemi) with 64 channels in a standard 10-20 configuration. This system uses a head cap system with pin active silver chloride electrodes. The EEG ground (labeled DRL [Driven Right Leg]) on the Biosemi system was placed between POz and PO4, and the EEG reference (labeled CMS [Command Mode Sense]) was placed between POz and PO3. The antialiasing filter was a fixed first-order analog filter with −3 dB at 3.6 kHz, and the low-pass filter was a fifth-order cascaded integrator-comb digital filter with −3 dB at one-fifth the sample rate. A powerline notch filter was not applied because the system used active electrodes, a battery power supply, and optic fiber, greatly reducing noise from the powerline. Each channel consisted of a 24-bit analog-to-digital converter. Recordings were made at 1024 Hz (although one was recorded at 2048 Hz) using ActiView software (Biosemi Instrumentation).

An HTC Vive VR system (HTC) with a Deluxe Audio Strap fitted over the top of the EEG cap was used. This system features a 2160×1200 resolution, 90 Hz refresh rate, and 110° field of view. Positional tracking from 2 infrared cameras enabled 5-degrees-of-freedom motion tracking of the headset and hand controllers. Integrated stereo headphones supported 3D audio immersions. During the initial pilot testing, this system was found to work effectively. Minor noise was evident in the EEG from the VR system on rare occasions, but we were able to remove most of this noise by careful repositioning of the equipment.

For meditation practice, a commercially available Guided Meditation VR application (Cubicle Ninjas) was used. As each meditation was only 10 minutes in this app, we selected a single sequence of 3 unique meditations, Zen 2-4, to form a 30-minute block of meditation. In the guided meditation experience, users were situated in the *Lost Woods* virtual environment with the *Calm* music selected. They were able to explore a calm, forest 3D environment with running water features with soft chirping bird and gentle wind sounds. Using a controller, participants could move to different positions in the forest to explore or find a particular viewpoint they liked and found most conducive to their meditation. A narrative provided audio guidance on the meditative practice. This environment was selected to maximize the similarity with the participants’ prior VR experiences in the RCT.

**Figure 1:**
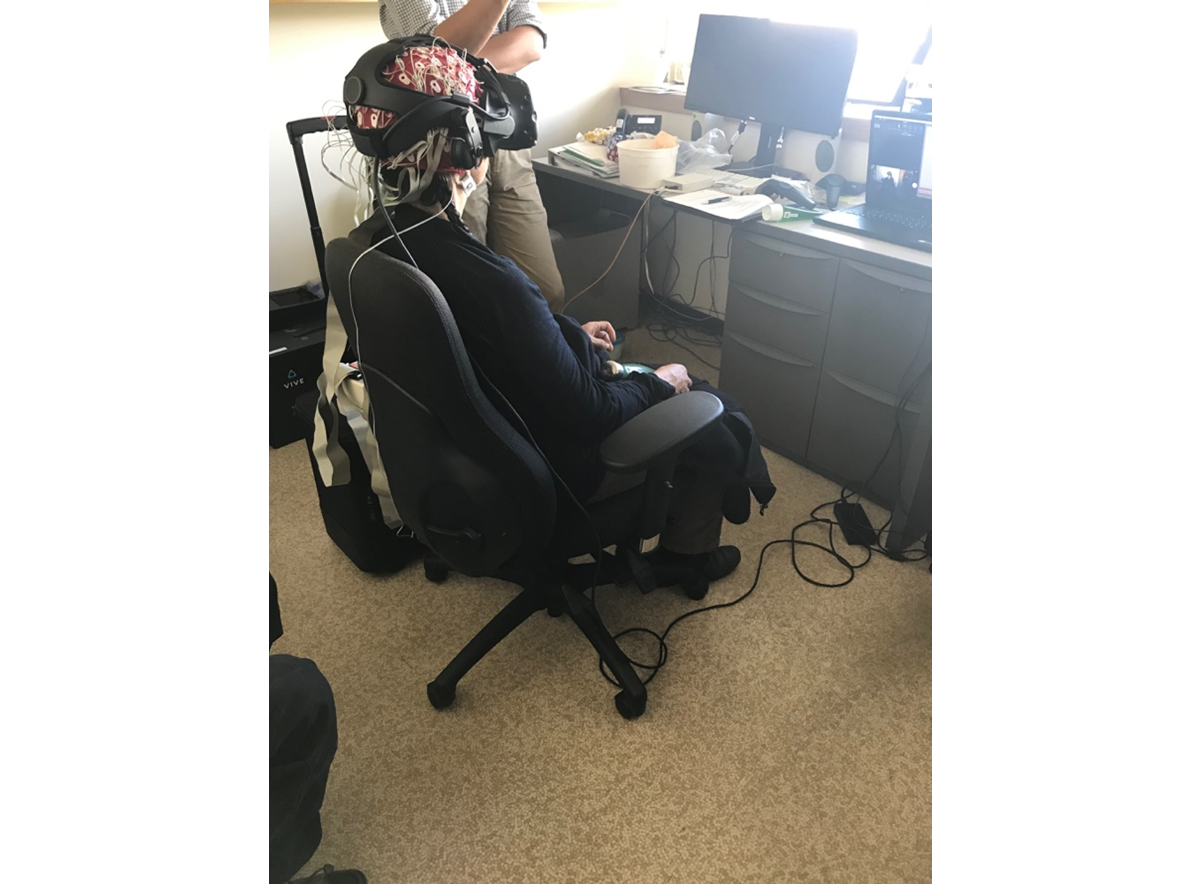
Electroencephalograph recording during a virtual reality experience.

Both EEG recording and the VR system were run on a Dell G7 17 7790 gaming laptop (Intel Core i7-8750H, 16 GB DDR4, RTX 2060) placed in front of the participant. During recording, the laptop screen was arranged to display the ActiView software and a mirror window of the Guided Meditation VR application experience, showing the participant’s perspective in the VR HMD. In addition, a Windows 10 (Microsoft) camera app was used to record the video. This arrangement was recorded using Snagit (Techsmith) screen capture software to accommodate the time synchronization of EEG and recordings of participants’ physical movements (Figure 1).

### Procedures

Participants were seated in front of the laptop, and the laptop webcam was framed to capture the participants’ head, arms, and upper body. Before putting on the EEG head cap, participants were familiarized with the VR app, its controls, and how to navigate and select meditations. The Cz, inion, and left and right preauricular locations were marked using standard EEG landmarking methods. These locations were used to align the EEG head cap on the participant. The electrode paste was then applied, and the electrode contacts were adjusted until the electrode offsets were less than 50 µV. The VR HMD was then placed in position over the EEG head cap, and the experience commenced.

**Figure 2:**
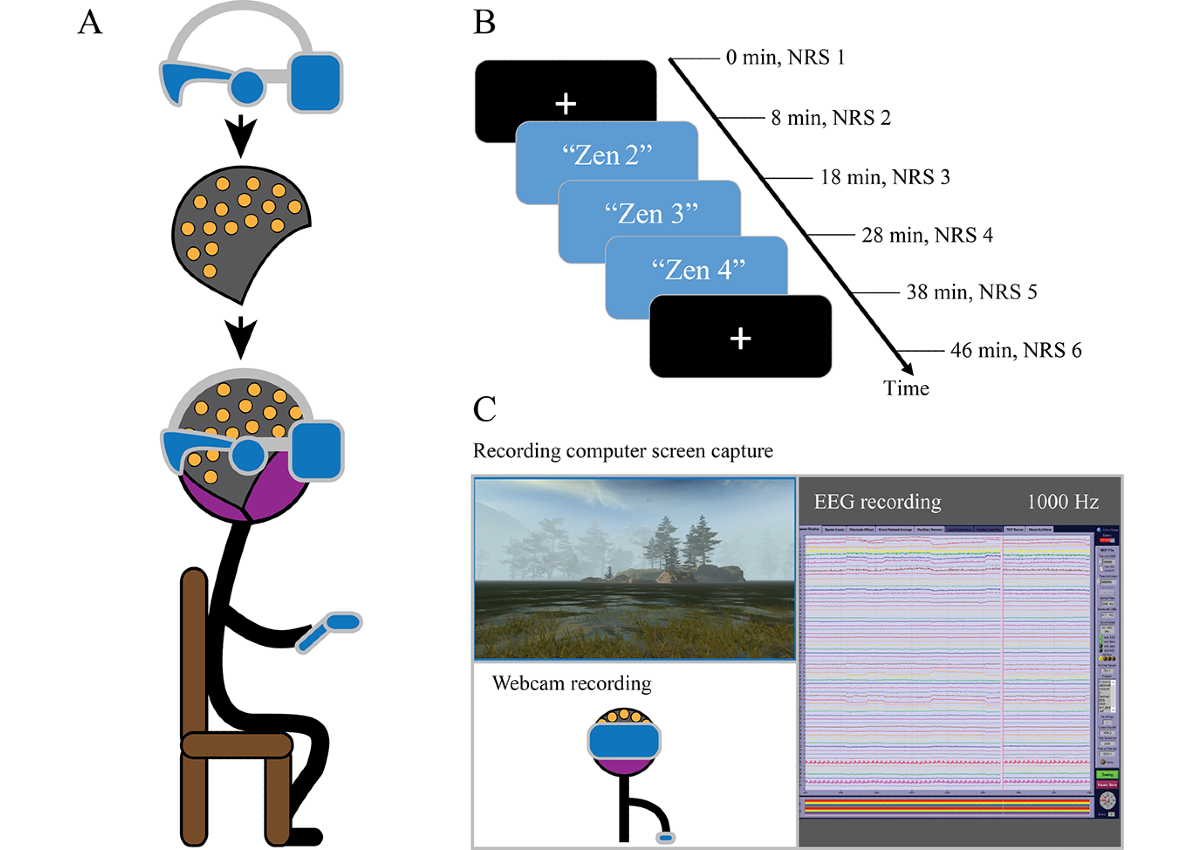
(A) Diagram of equipment setup. (B) Timeline of recording, including rest and meditation conditions. (C) Diagram of desktop view. EEG: electroencephalograph; NRS: numerical rating scale.

The overall structure of data collection used an 8-minute resting condition, followed by the 30-minute sequence of meditations and followed by another 8-minute rest condition (Figure 2). The 8-minute period was considered a balance between being shorter to reduce strain and being longer for more data; this period has been used in EEG studies in a variety of fields for baselines [49-51].

During the rest conditions, the participants were asked to rest quietly and observe a small white crosshair displayed on a black background in the VR HMD. Participants were instructed to keep their eyes open while blinking naturally, to keep their eyes on the crosshair, and to stay still while not thinking about anything. During the meditation practice, participants were instructed to engage with the guided meditation and look around or move around the virtual environment as they liked, to find an enjoyable perspective. Each guided meditation experience lasted 10 minutes, and participants were instructed to begin the next one immediately after the intervening rest condition. Pain was assessed before and after the first rest condition, after each 10-minute guided meditation, and after the second rest condition. Participants were asked to verbally rate their pain from 0 to 10 on the simple numerical rating scale (NRS) [52].

### Analysis

#### Data Preprocessing

The recording sessions of the resting condition, the 3 guided meditation conditions, and the final resting condition were referred to as the *Pre*, *Med1*, *Med2*, *Med3*, and *Post*, respectively, for the analysis. These 5 conditions were extracted based on the time stamps acquired from the EEG video recordings.

**Figure 3:**
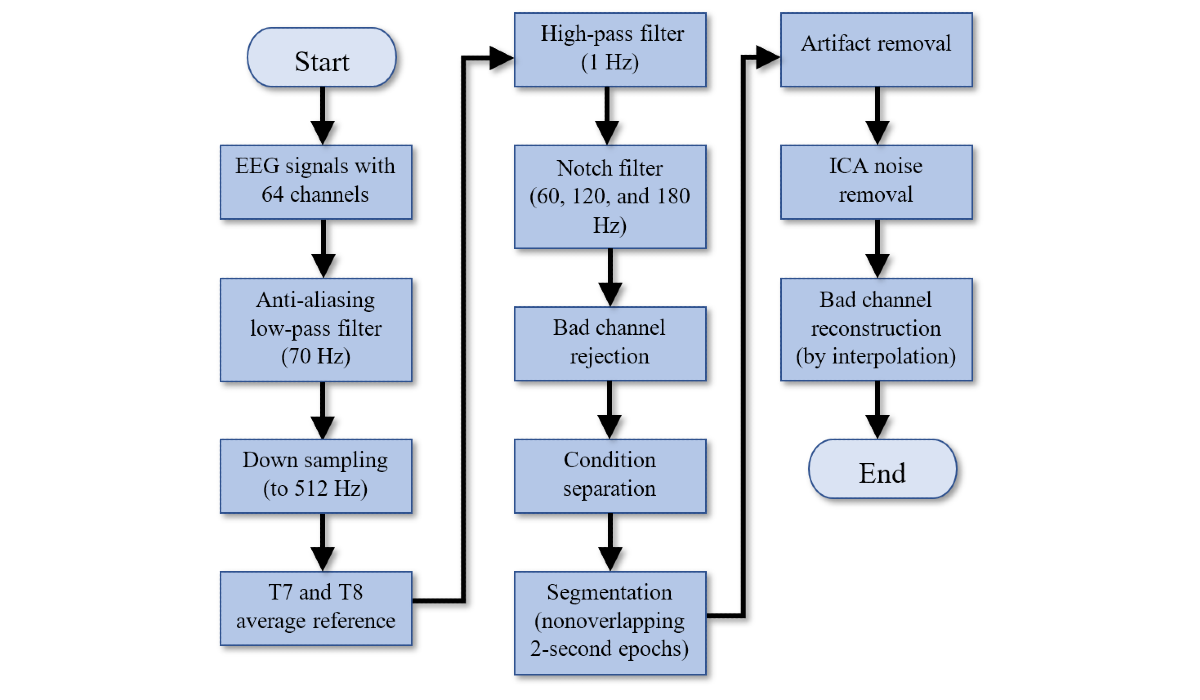
Electroencephalograph data preprocessing chart. EEG: electroencephalograph; ICA: independent component analysis.

Figure 3 illustrates the EEG data preprocessing steps. The raw EEG data in the Biosemi format were imported and preprocessed using FieldTrip software (Donders Institute for Brain, Cognition and Behaviour) [53]. As the bandwidth of interest was less than 50 Hz, the data were downsampled to 512 Hz to reduce computing time in later processes. To prepare for downsampling, an antialiasing sixth-order low-pass Butterworth two-pass filter set to 70 Hz was first applied to the data. The primary 64 EEG channels were then re-referenced to the averaged left and right mastoid electrodes (T7 and T8). This re-referencing process effectively eliminated the noise introduced from the original reference electrodes. After this process, the reference electrodes T7 and T8 were eliminated from the data, and only 62 channels were retained.

In addition, a sixth-order high-pass Butterworth two-pass filter with a cut-off at 1 Hz was used to remove slow drifts and to prepare the data for independent component analysis (ICA) [54]. The data were further notch-filtered to remove any powerline noise and harmonics, with cut-offs set at 60, 120, and 180 Hz. Bad channels were identified during the EEG recording process using an EEG data browser and rejected.

These data for each condition were further segmented into nonoverlapping epochs of 2 seconds in length to enable signal averaging in the frequency domains [55]. A data cleaning process was then performed using the FieldTrip automatic artifact removal feature, which is based on a Z-transformation and the setting of a threshold to reject bad epochs. This artifact removal result was found to be unsatisfactory, so a manual cleaning process was then performed. Segments with participant movement observed in the videos were identified, and any residual bad segments and channels were rejected. These steps effectively eliminated the artifacts caused by head and body movements.

Any remaining artifacts caused by eye blinks, eye movements, and external noise were eliminated from the data using ICA. These manual artifact removal and ICA processes were repeated 3 times during the cleaning process to ensure that all movement-based artifacts had been captured. Finally, bad channels were repaired [56] using the established practice of spline interpolation of the neighborhood channels on the bad channels [57].

#### Power Spectral Density Analysis

The power spectral density (PSD) [58] was computed for the conditions using a fast Fourier transform method [59]. Hanning taper smoothing was applied to reduce spectral leakage owing to the discontinuity of the signal at the start and end points of the epochs. To improve the signal-to-noise ratio, the average PSD of the epochs was used. The PSD was further normalized by dividing the average power across all the frequency bins in each channel. The frequency range in the analysis was set to 2-50 Hz, which effectively covers the waveforms commonly used in EEG analysis. The PSD results of the premeditation experiences, during meditation experiences, and postmeditation experiences were then plotted and shown as pre, med, and post conditions, respectively. For med, the average PSD of the meditation conditions was computed using the average of the 3 meditation conditions (Med1, Med2, and Med3). For a graphical overview, a band power box and whisker plot were created. The plot revealed the signal power over the typical EEG frequency bands: 2-4 Hz (δ), 4-8 Hz (θ), 8-12 Hz (α), 12-30 Hz (β), and 30-50 Hz (γ) [60]. The lower boundary of 2 Hz was set to avoid any distortion introduced by the high-pass filter at 1 Hz. The upper boundary of 50 Hz was selected to avoid the heavy power-level drop due to the use of a 60 Hz notch filter. In addition, 50 Hz was considered a reasonable cut-off between the high and low γ ranges.

#### Topographic and Coherence Analysis

To explore the spatial properties of the signals on each EEG channel, a topographic mapping of the PSD was plotted according to the traditional frequency ranges. The PSD was plotted using a heat map visualization technique to display the magnitude of the PSD with a 2D color representation. An interpolation based on the MATLAB 4 grid data method was used to smooth the topography, where a surface was fitted to the scattered data points using a biharmonic spline interpolation [61]. This PSD plot was used to graphically illustrate the spatial properties associated with the pre, med, and post experiences.

A coherence analysis [62] was performed to visualize the functional connectivity between the electrodes as an indication of the brain areas that may be functionally integrated. Coherence of the pre, med, and post conditions were plotted for the frequency bands. A value between 0 and 1 was displayed for each channel pair. A value of 1 indicates full synchronization between the channel pair, and a value of 0 reveals that the channel pair does not work in the synchronized condition. The significance of the coherence difference between the conditions was computed using the two-tailed Student *t* test and then plotted.

#### Pain

Pain experience was measured using the NRS. Pain scores were collected from the patients as an initial baseline at the start, and after each condition, to explore any correlation between pain levels and any PSD changes identified during the meditative experience.

#### Statistical Analysis

For the PSD and coherence analysis, the null hypothesis set for inferential statistical analysis was that the probability distribution of the condition-specific averages for PSD and coherence would be identical for all conditions.

As a cluster-based permutation test [63] can be used to effectively resolve multiple comparisons in EEG signal statistical analysis, this test was used in this study to examine the overall PSD differences between the pre-med, med-post, and pre-post conditions by identifying the clusters of electrodes with significant changes. The tests were conducted using the Monte Carlo method, with 128 permutations, two tails, and α=.025 (negative and positive tails together equal .05). In addition, for comparison of all 3 conditions together, a multivariate analysis of variance (MANOVA) was used, with 2048 permutations, one tail, and α level set to .05.

The effect size was calculated after the cluster-based permutation test. First, a bounded rectangular area spanning each cluster was identified. This rectangular area was bounded by the frequency window of the cluster and all the channels in that cluster. The PSD in this area was then averaged. The maximum Cohen *d* effect size was then computed using FieldTrip for each of the conditions. The effect size was not computed for the MANOVA because it requires the specification of 2 mean groups for comparison. Generally, a Cohen *d* effect size around 0.2 is considered small and around 0.8 and higher is considered large to very large [64,65]. Finally, peak channel tests were performed to verify the PSD changes, and post hoc power analyses using G*Power [66] were performed to indicate statistical power based on the effect sizes observed in the sample.

For the coherence analysis, the Student *t* test was used to examine significant coherence differences between the conditions. Parameters were set to two-tailed, paired samples, and α=.05, with a 10% false discovery rate (FDR) adjustment set [67].

To explore the associations between pain scores and PSD, repeated measures correlation [68] was conducted. Some peak PSD channels identified with a high significance of PSD change from the cluster topoplot were input for each condition as the repeated independent variable, and the NRS pain score was input as the dependent variable.

## Results

### Overview

A total of 10 participants were recruited (Table 1). As we went through the data cleaning process, the data from 1 participant (S05) were found to be excessively noisy, and on video review, 2 other participants (S08 and S09) were found not to be following the procedures. Hence, their results were excluded, and a sample size of 7 was used in the final analysis.

**Table 1:** Participant demographics.

Subject	Age (years)	Sex	Cancer history
S01	59	Female	Abdominal tumors
S02	66	Male	Non-Hodgkin lymphoma
S03	37	Male	Non-Hodgkin lymphoma
S04	58	Female	Breast cancer
S06	47	Female	Chondrosarcoma
S07	50	Female	Colon cancer
S10	64	Male	Prostate cancer

### PSD Analysis

**Figure 4:**
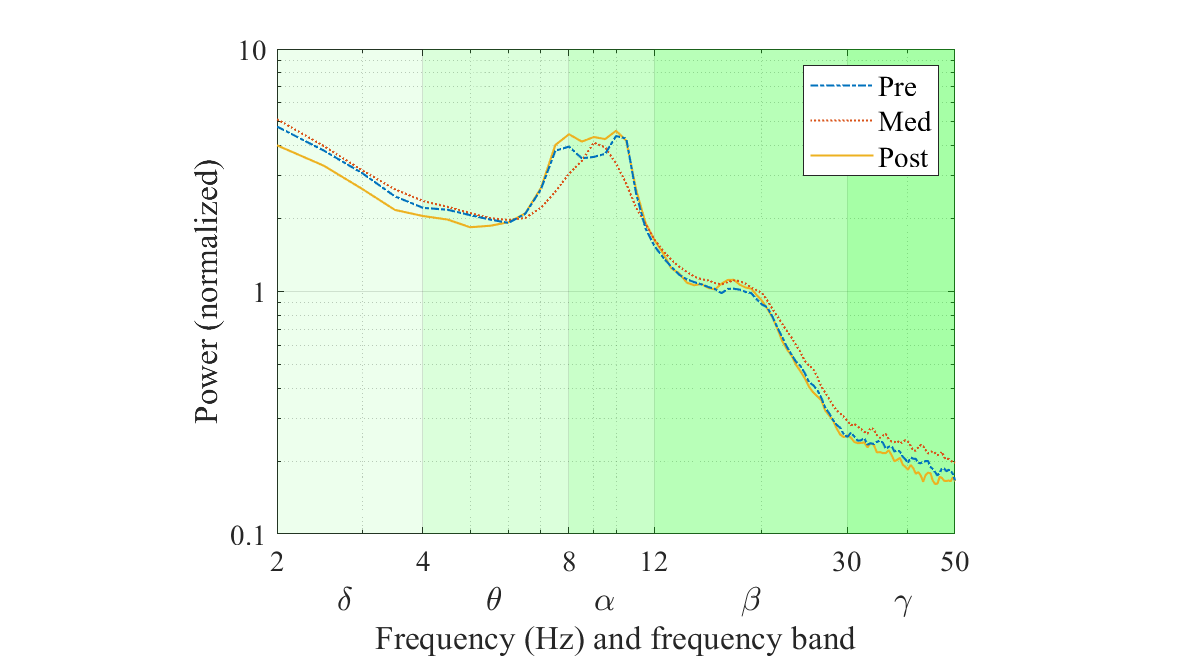
Grand average power spectrum.

**Figure 5:**
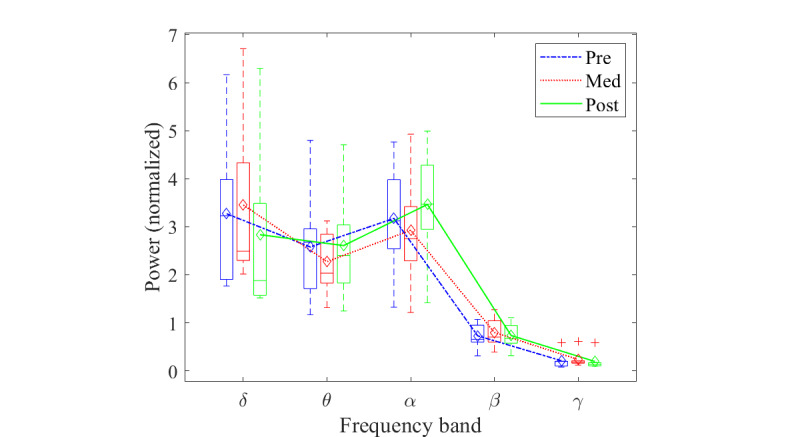
Power level changes between the pre, med, and post conditions in different frequency ranges. Box plot shows the median and range of power level of the participants. Line plot shows the changes in mean power.

Figure 4 illustrates the average of the power spectrum of the 3 conditions to provide a general overview of the power spectrum in the data, whereas Figure 5 shows the box and whisker plot of band power of the pre, med, and post conditions in the 5 traditional frequency ranges. From these figures, the differences in power in the frequency bands between the conditions were identified. The post condition power level had the lowest median and mean values in the δ range. The med condition changed from the lowest median and mean values in θ and α to the highest median and mean values in β and γ.

**Figure 6:**
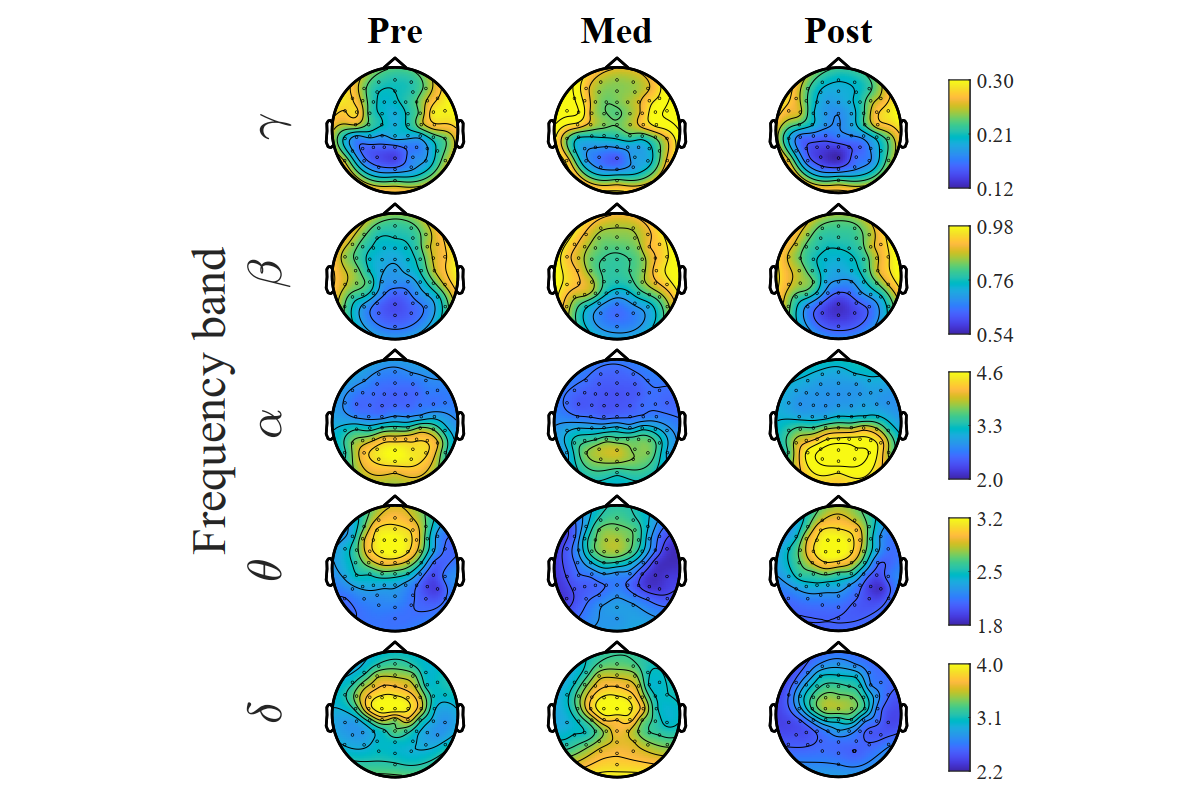
Topography of power spectrum shown in different frequency ranges. Different color scales are used for the frequency ranges to reveal the details in the central areas.

The topographic distribution of the PSD is shown in Figure 6. Differences in pre, med, and post conditions are shown spatially in all bands and in different brain regions. Changes in the power levels during meditation were observed in all frequency bands. In δ, an increase in power level in the central occipital region was observed, and a drop of power in the frontal-central region was observed in the post condition. In θ, there was a drop in the power level in the frontal cortex. In α, a decrease in power level in the central parietal region was noted. In the β band, an increase in power level was found in the bilateral central and prefrontal regions during meditation. In γ, an increase in power level was noted in the left frontal (LF) and right frontal (RF) regions, and a slight increase in the central parietal region was observed.

**Figure 7:**
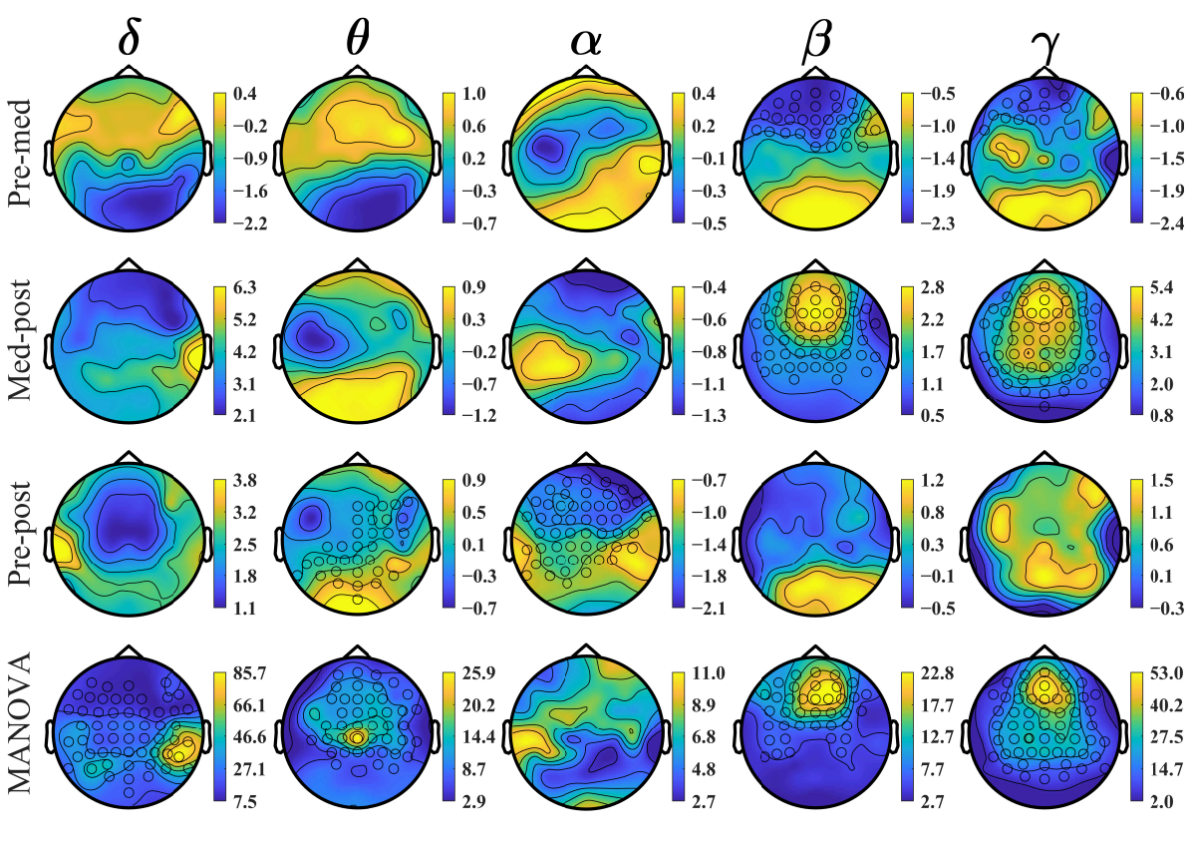
Power spectrum analysis using the cluster-based permutation test. Clusters of electrodes found with significant changes in power are marked with a circle marker at the electrodes. The color bars show the permutation test t-value level. MANOVA: multivariate analysis of variance.

Figure 7 shows the topoplot of the two-tailed cluster-based permutation test results. The conditions were compared in the test, including two-tailed pre versus med, med versus post, and pre versus post and a one-tailed MANOVA for all 3 conditions together. The clusters of electrodes with significant differences found in PSD changes were marked with a circle marker at the electrodes. The color bars indicate the *t* values computed from the test. For the pre versus med comparison, a cluster with a significance of *P*=.02 was found in the range of 24.5-31 Hz (high β and low γ) in the frontal cortex. For the med versus post comparison, significance was found in the β and γ ranges. A cluster with a significance of *P*=.001 was found in the range of 37-50 Hz (γ), and the cluster covered most brain regions; a cluster with a *P* value of .008 was found in the range of 23-36 Hz (high β and low γ) in the frontal and central cortices. For the pre versus post condition comparison, a cluster with *P*=.02 was obtained in the frontal, central, and parietal regions in the range of 8-9.5 Hz (high θ and low α). The MANOVA cluster-based permutation test for all the pre, med, and post conditions returned 4 clusters with *P*≤.05, where the test was one-tailed. The first cluster found has *P=*.002 in the range of 37.5-50 Hz (γ) in the frontal, central, and parietal cortices. The second cluster with *P*=.03 was found in the range of 31-36 Hz (low γ) in the frontal and central cortices. The third cluster with *P*=.03 was found in the range of 2-5 Hz (δ and low θ) in the frontal, central, and parietal cortices. The last cluster with *P*=.04 was found in the range of 24-30 Hz (high β) in the frontal and central cortices. Table 2 shows the test results for all the clusters of significance. The very large effect size indicated that the permutation tests were effective in rejecting the null hypothesis.

**Table 2:** Cluster-based permutation test results. All clusters with P≤.025 are shown for the first 3 tests, and clusters with P≤.05 are shown for the multivariate analysis of variance test.

Comparison and cluster			*P* value		Effect size
**Pre-med**					
	1	.02		1.252	
**Med-post**					
	1	.001		3.190	
	2	.008		1.318	
**Pre-post**					
	1	.02		1.504	
**MANOVA^a^**					
	1	.002		—^b^	
	2	.03		—	
	3	.03		—	
	4	.04		—	

^a^MANOVA: multivariate analysis of variance.

^b^Not calculated as it required to specify which 2 conditions to compare.

By inspecting the topoplots (Figures 6 and 7), 5 peak channels with noticeable changes in the power levels between the conditions were selected for additional analysis and for better understanding the observed power level changes. The peak channels selected were AF7 and Fp2 in the prefrontal region, FC1 in the frontal region, CP5 in the left central (LC) region, and P5 in the left parietal (LP) region. Two-tailed, paired-sample *t* tests were performed to examine the overall PSD changes in different conditions. These tests were conducted on the pre and med, med and post, and pre and post conditions. The resulting *P* values were reported with an FDR adjusted to 10%. The *t* test results were plotted graphically for ease of interpretation. For each of the peak channels, a combined plot of the results of the 3 conditions was used.

**Figure 8:**
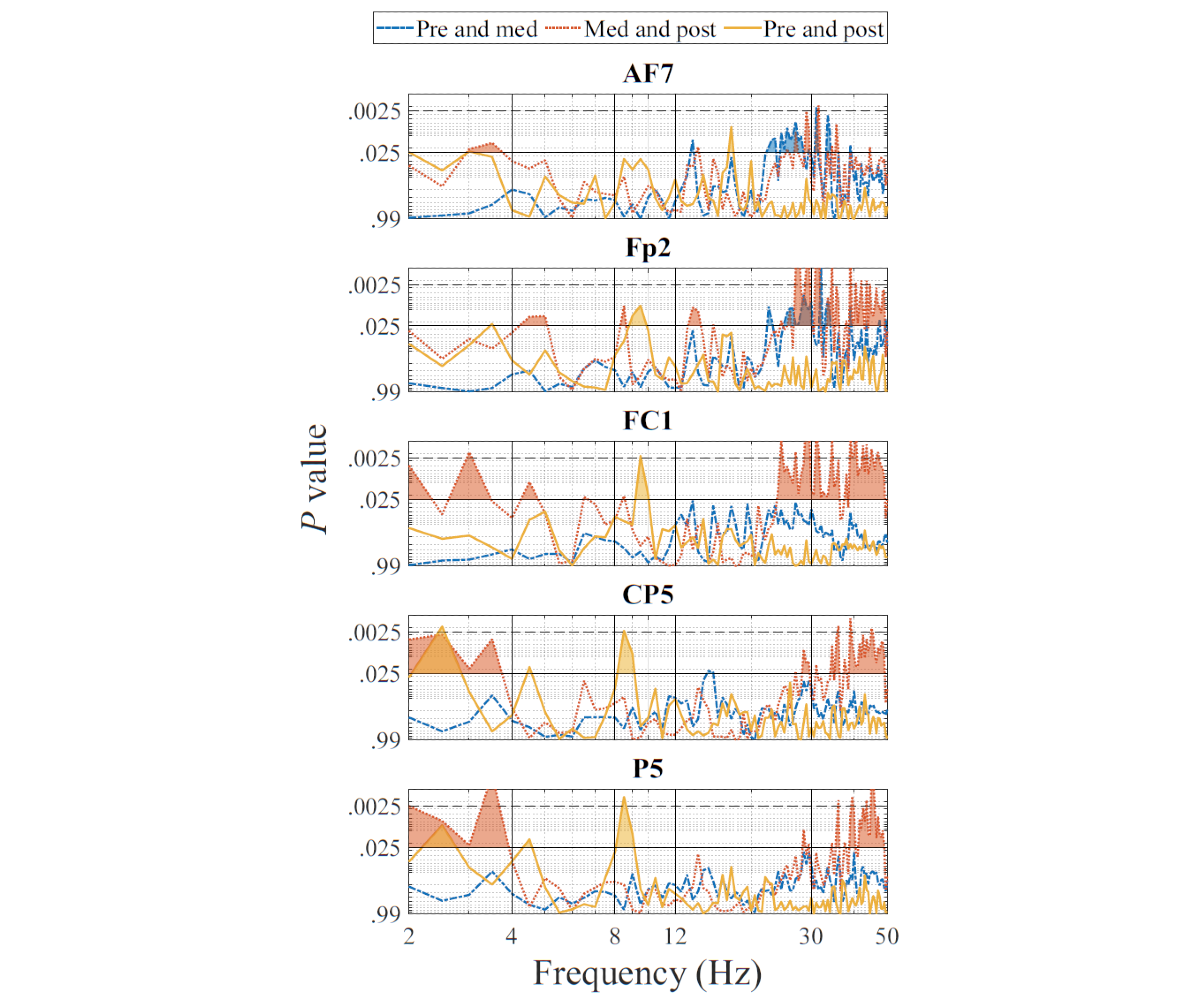
Graphical depiction of *P* values of changes in power spectral density between conditions for the selected channels. Shading indicates significance found at the .025 level, and shading above the dashed line indicates the adjusted significance at the .0025 level (with the false discovery rate set to 10%).

Figure 8 shows the *P* values obtained in a graphical form for the comparison of the 3 conditions. The upper shaded areas represent *P*≤.025. The shaded areas above the dashed line represent the adjusted significance with *P*≤.0025.

A statistical power analysis was also performed to verify the probability of detecting a true effect in the *t* tests. Table 3 shows the maximum effect size value and the respective frequency point found for each channel and for each comparison. Table 4 shows the statistical power based on the effect sizes listed in Table 3. It is shown that the *t* test results have a high power ≥0.7302 even when using the adjusted α value of .005.

**Table 3:** The maximum channel effect size and the frequency at which it was located.

Channel (N=7)	Pre-med			Med-post			Pre-post	
	Max effect size	Frequency (Hz)	Max effect size		Frequency (Hz)	Max effect size		Frequency (Hz)
AF7	*1.9542^a^*	*31*	*1.9946*		*31.5*	1.5613		17.5
Fp2	*2.3861*	*32*	*3.7903*		*31.5*	1.4682		9.5
FC1	1.1037	13.5	*2.9888*		*29*	*1.9221*		*9.5*
CP5	1.1651	15	*2.1765*		*39*	*1.9937*		*2.5*
P5	1.0514	40	*2.6077*		*3.5*	*2.0736*		*8.5*

^a^Italicized values indicate where *P*≤.0025 was found for that frequency value.

**Table 4:** Power analysis for the effect size found.

Channel	Pre-med, α=.005	Med-post, α=.005	Pre-post, α=.005
AF7	*0.7472^a^*	*0.7676*	0.5073
Fp2	*0.9125*	*0.9998*	0.4450
FC1	0.2225	*0.9890*	*0.7302*
CP5	0.2556	*0.8470*	*0.7672*
P5	0.1962	*0.9558*	*0.8047*

^a^Italicized values indicate where *P*≤.0025 was achieved.

### Topographic and Coherence Analysis

**Figure 9:**
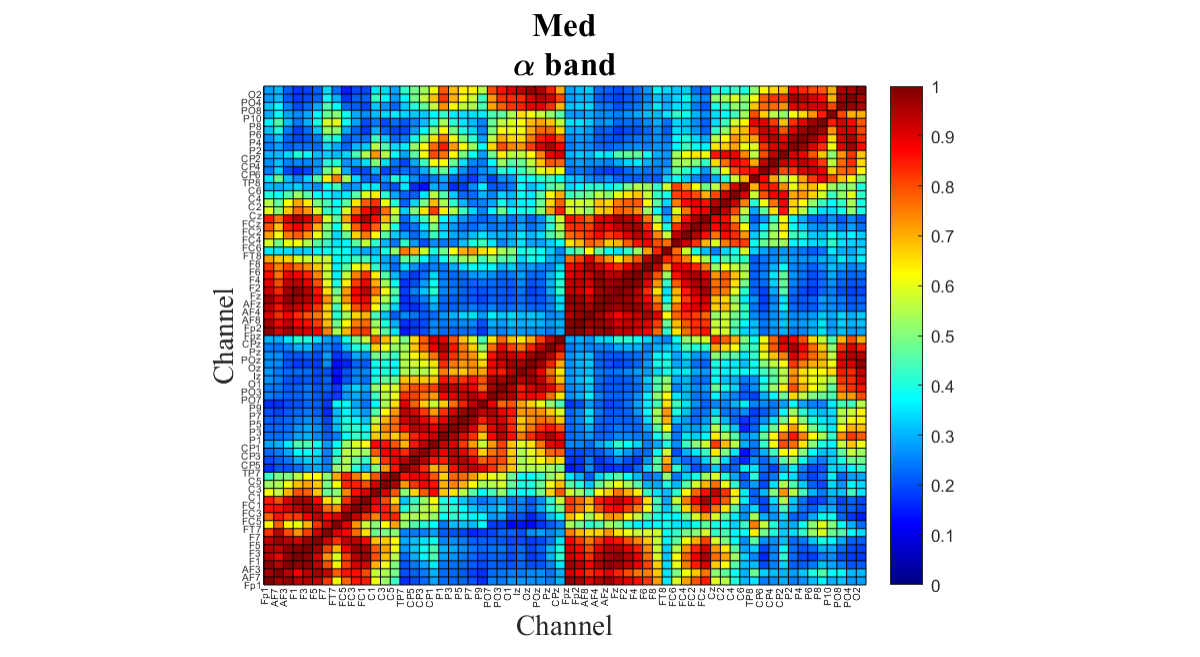
Coherence between the electroencephalograph channels during the virtual reality–guided meditation and in the α frequency range.

Figure 9 demonstrates the coherence detected in the α band during meditation.

The functional connectivity level is represented by a red to blue color scale. The red color with a value close to 1 indicates that a channel pair is highly synchronized in the signal transfer. The blue color with a value close to 0 indicates that the channel pair is working independently.

**Figure 10:**
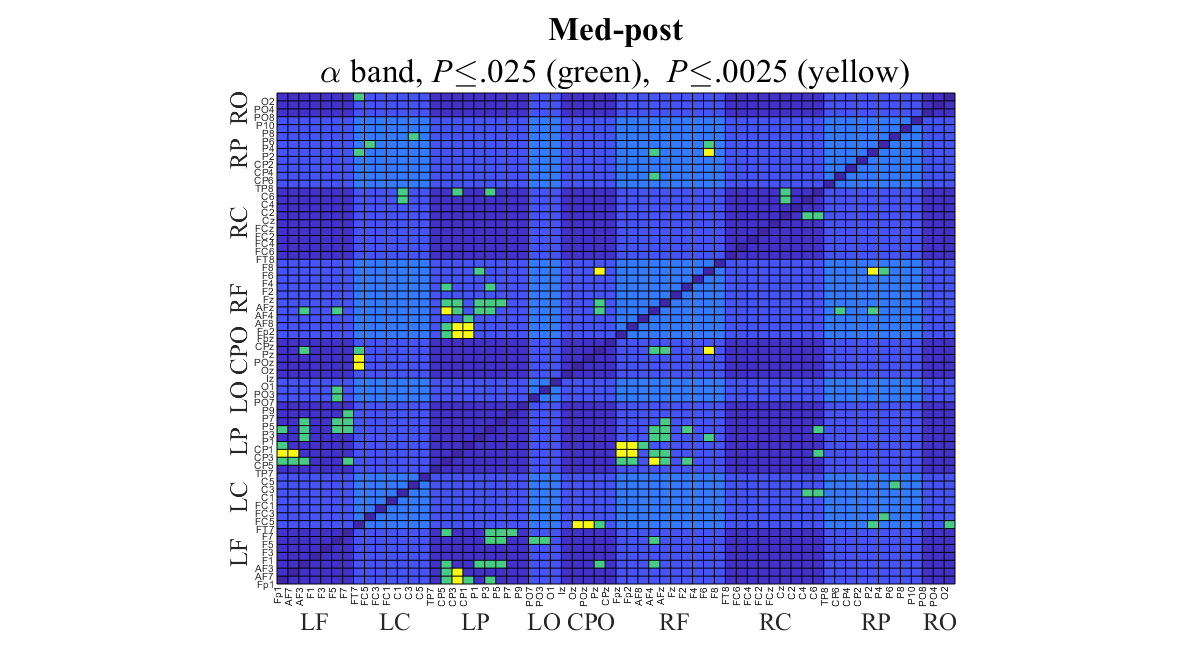
Channel pairs with significant coherence difference between the meditation and post meditation conditions in the α band. t test was used in the analysis, and channel pairs with a P≤.025 are highlighted in green, and channel pairs with a P≤.0025 (with the false discovery rate set to 10%) are highlighted in yellow. CPO: central parietal and occipital; LC: left central; LF: left frontal; LO: left occipital; LP: left parietal; RC: right central; RF: right frontal; RO: right occipital; RP: right parietal.

Figure 10 shows the significant difference in coherence for the comparison of the med and post conditions in the α band. Channel pairs with *P*≤.025 are highlighted in green. Those with *P*≤.0025 (FDR adjusted) are highlighted in yellow. For clarity, the EEG channels were divided into regions according to their respective locations in the brain, namely, LF, LC, LP, left occipital, central parietal and occipital, RF, right central, right parietal and right occipital. Table 5 summarizes the results of a series of plots, as shown in Figure 10, for all the bands and all 3 comparisons. The table shows the significant coherence changes with *P*≤.0025 between the brain regions, where 1 denotes the pre and med comparison, 2 denotes the med and post comparison, and 3 denotes the pre and post comparison. The frequency band of the region pair at which a significance was found is shown with the Greek letter of the band. The number inside parentheses indicates the number of significant channel pairs in the frequency band.

**Table 5:** False discovery rate–adjusted significant coherence changes found in the region pairs for the 3 comparisons: 1: pre versus med, 2: med versus post, and 3: pre versus post.

Region	LF^a^	LC^b^	LP^c^	LO^d^	CPO^e^	RF^f^	RC^g^	RP^h^	RO^i^
RO	3: δ^j^	3: θ	—^k^	—	—	—	—	—	✓^l^
RP	—	—	2: δ(2)^m^	2: δ	2: δ	2: α; 3: β	1: θ	✓	—
RC	—	—	—	—	—	1: β	3: α (✓)	—	—
RF	—	—	1: γ(2); 2: θ(2), α(5); 3: δ(2)	—	2: α	✓	—	—	—
CPO	3: δ α	1: α; 2: α(2)	—	—	✓	—	—	—	—
LO	—	—	—	✓	—	—	—	—	—
LP	2: θ, α(2), β	—	✓	—	—	—	—	—	—
LC	—	✓	—	—	—	—	—	—	—
LF	✓	—	—	—	—	—	—	—	—

^a^LF: left frontal.

^b^LC: left central.

^c^LP: left parietal.

^d^LO: left occipital.

^e^CPO: central parietal and occipital.

^f^RF: right frontal.

^g^RC: right central.

^h^RP: right parietal.

^i^RO: right occipital.

^j^Greek letter indicates the frequency band of the region pair with significant change.

^k^The empty cells in the upper left of the check mark diagonal show no significant channel pair was found. The cells in the lower right of the check mark diagonal are not used, as they are just mirrored duplicates of the cells in the upper left of the check mark diagonal.

^l^The checkmark indicates a region connects to the same region in the region pair.

^m^Number within parentheses indicates the number of channel pairs with significant changes in that frequency band. If there is only 1 channel pair, the number is not shown.

As shown in Table 5, significant changes were mostly found between the frontal and parietal regions, namely, RF-LP (11 channel pairs) and LF-LP (4 channel pairs). In addition, the most active regions were LP (related to 17 channel pairs), RF (15 channel pairs), LF (7 channel pairs), right parietal (7 channel pairs), and central parietal and occipital (7 channel pairs). For the 3 comparisons overall, significant changes were mostly found between the frontal and parietal and occipital regions, particularly in the θ, α, and β bands in the med-post comparison. For the pre-med comparison, the frontal-parietal region-pair coherence changes were observed in the γ band. For the pre-post comparison, the frontal-parietal and occipital region-pair coherence changes were observed in the δ, α, and β bands. Table 6 shows the effect sizes of the 2 regions of interest, namely, RF-LP and LF-LP. Some large to very large effect sizes (≥0.8) were found in the δ, θ, α, and γ bands.

**Table 6:** Effect sizes of the 2 region pairs of interest: right frontal-left parietal and left frontal-left parietal.

Band	Region-pair RF-LP^a^ average, effect size				Region-pair LF-LP^b^ average, effect size		
	Pre-med	Med-post	Pre-post	Pre-med		Med-post	Pre-post
δ	0.2873	0.7545	*0.8511^c^*	0.1636		0.4461	0.2862
θ	0.5568	0.7738	0.1496	0.7464		*0.8234*	0.1610
α	0.5855	*1.3032*	*0.8593*	0.7742		*1.7736*	*1.0655*
β	0.4643	0.6142	0.2211	0.2759		0.5135	0.1294
γ	0.3482	*1.0220*	0.2218	0.3032		0.5880	0.1633

^a^RF-LP: right frontal-left parietal.

^b^LF-LP: left frontal-left parietal.

^c^Italicized values indicate large and very large effect sizes.

Table 7 shows the NRS pain scores collected immediately after the pre, Med1, Med2, Med3, and post conditions. A repeated measures correlation analysis was performed on some selected peak channels and clusters. In the test, no significant relationship between the collected pain scores and PSD was found.

**Table 7:** Numerical rating scale scores collected after each condition.

Participant	NRS^a^ score				
	Pre	Med1	Med2	Med3	Post

S01	4	2	2	2	1
S02	4	3	0	0	1
S03	7	4	7	5	3
S04	6	5	4	4	3
S06	5	6	5	4	5
S07	3	5	4	2.5	4
S10	3	3	2	2	2

^a^NRS: numerical rating scale.

## Discussion

### Principal Findings

#### Overview

In answer to the primary research question, there were significant changes in EEG power and coherence among three conditions (pre, VR-guided meditation, and post). Therefore, the null hypothesis of no difference was rejected.

The visual inspection of the global normalized power spectrum analyses revealed various changes in all bandwidths. The predominant pattern was for increased δ, β, and γ bandwidth power in the meditation condition, compared with both the pre and post conditions. In the θ and α bandwidths, the changes in power were more varied within the 3 conditions.

#### Pre Medication Versus VR-Guided Meditation

Visual inspection of the topographic distribution showed 2 main patterns comparing meditation with the prior resting condition. The first was an increase in power of δ (mainly in the central and occipital areas), β (mainly in the bilateral prefrontal areas), and γ (mainly in the frontal and bilateral prefrontal areas) during VR-guided meditation.

The second pattern that emerged from the visual topography map was decreased low-frequency range power of the θ (mainly in frontal areas) and α (mainly in occipital and parietal areas) bandwidths in the med condition compared with the pre condition. However, these visually observed changes were not significant in the permutation test.

Among the significant changes identified, the permutation test showed that a cluster of increased signals occurring across the high β and low γ range (24.5-31 Hz) in frontal areas was significantly different in the pre condition than in the med condition. In addition, comparison of single selective channels between conditions showed a significant difference in this bandwidth in the frontal areas recorded from AF7 and FP2.

β waves generally replace α waves when participants open their eyes, and in the motor cortex, β waves are associated with muscle motor activity [58,60]. They are normally most prominent in the frontal and central head regions and attenuate posteriorly. This may have been the case for the increase observed here. β activity is also commonly associated with drowsiness, stage nonrapid eye movement 1 sleep, and subsequently decreases in deeper sleep, and β activity is not affected by eye opening [60]. Interestingly, sedative medications are also known to increase the amplitude and quantity of β activity [69]. This finding suggests that an increase in the power of the β range might potentially be useful as a neurophysiological correlate of VR-guided meditation. Nevertheless, findings regarding changes in the β band with meditation have been inconsistent. Several studies have reported no significant changes associated with meditation in the β range [4,31]. However, an increase in β and θ band power was reported in one study after a longer period of 6 weeks of meditation compared with baseline [70]. It is possible that the changes in β power in the meditation condition compared with the premeditation condition here may be more specific to the use of VR-guided meditation and the visual activity involved, but further work is required to explore this.

In contrast, the activity of the γ band has been reported to be associated with activation of the default mode network. The default mode network is most frequently detected during the resting conditions and reflects the neural activity of different brain areas, such as the cingulate cortex, hippocampus, medial frontal lobes, inferior parietal lobes, and temporal lobes. It is thought to be involved in self-consciousness; self-processing; and introspective functions, including emotional awareness and processing [71-74]. An increase in γ band activity in the frontal and prefrontal areas during VR-guided meditation could reflect the activation of such introspective experiences through meditation. The increase in γ power may also have been due to the activation of attentional networks and visual processing of the meditative VR environment [75].

It is also noteworthy that others have reported an increase in γ band power related to meditation [17,75,76]. For example, one study reported an increase in the γ band (25-45 Hz) during meditation compared with the resting state in the temporal and parieto-occipital areas in mindfulness meditation practitioners [76]. Another study reported that the proficiency level of the meditator is associated with the increased level of the γ band (60-110 Hz) in the parieto-occipital region in meditative states relative to the mind-wandering state in experienced meditators compared with healthy controls. Although it is noteworthy, in this study, no significant difference was found between the states of meditation and mind wandering [75].

#### VR-Guided Meditation Versus Post Meditation

In contrast to the pattern of increase in high β and low γ activity in the meditation condition compared with the prior rest condition, here, we observed a pattern of reduced β and γ power in the post condition compared with the meditation condition in the topographic maps. This was followed by permutation test results in terms of 2 significant clusters of differences in the β cluster (23-36 Hz) and γ cluster (37-50 Hz) in the post versus med condition. These findings suggest a potential regression back to the baseline state activity and further suggest that changes in high β and low γ activity are associated with a VR meditative state. Analysis of single selective channels also supported widespread significant differences in the power of β and γ bandwidths in the frontal, central, and parietal channels (FP2, FC1, CP5, and P5).

As γ band oscillation has also been reported to be associated with attention toward pain and hypervigilance [77,78], the significant reduction in γ band activity following the VR meditation experience could potentially show that less attentional capacity is directed toward pain after using a meditative VR environment. However, this is conjectural and requires further verification.

#### Pre Meditation Versus Post VR-Guided Meditation

The comparison of the pre and post conditions could provide an indication of a VR-guided meditation effect in our study. These changes were mainly observed in the α frequency range in terms of an increase in α power in the frontal and central areas in the post condition compared with the pre condition. This was accompanied by a significant cluster-based permutation analysis finding over a cluster of channels in the frontal and central areas (8-9.5 Hz) and significant differences in α power in channels such as Fp2, FC1, CP5, and P5. An increase in the θ band in the central areas and a decrease in the θ band in the posterior occipital areas were also observed in the power spectrum analysis; however, these changes were not found to be significant in single-channel analysis. The posterior dominant α rhythm is characteristically present in normal conscious EEG recordings in the occipital region. It is a defining feature of the normal background rhythm of the adult EEG, best observed with the eyes closed and during mental relaxation and is attenuated by eye opening and mental effort. θ waveforms are characteristically observed more in drowsiness and in the early stages of sleep, such as light sleep (the nonrapid eye movement 1 and nonrapid eye movement 2 sleep phases) [60]. Increases in α-θ bandwidths have previously been reported to be associated with mindfulness meditation, and the α-θ border (7-8 Hz) has also been suggested as an optimal range for indicating visualization activity [4,79,80].

A final observation worthy of note is that there was a reduction in the δ range power in the post condition compared with the pre condition, which was significantly different in the LC and parietal channels (CP5 and P5). Moreover, although changes in the δ range were not significant in the pairwise pre-post comparison, a cluster of channels was identified to be significant in MANOVA. δ is seen more in deep, dreamless sleep, and meditation activities where awareness is more detached [4,60]. Such a general pattern of reduction in δ power in the post condition compared with the pre condition could possibly be related to the effect of VR-guided meditation on brain activity and would need further work to explore if this is a significant trend.

#### Coherence

The significant coherence changes suggested that variations in brain connectivity occurred between the different test conditions. Coherence was found predominantly between the frontal and parietal and occipital cortices and in different wave bands, namely, in γ for the pre and med conditions; in θ, α, and β for the med and post conditions; and in δ, α, and β for the pre and post conditions. The γ coherence changes between the pre and med conditions were likely associated with activity during the VR-guided meditation. Between the med and post conditions, it shifted to slower frequencies, possibly suggesting a postmeditation effect. The pre-post comparison showed coherence in the δ, α, and β ranges. The reasons for this are unclear and could be related to the individual responses of the participants after the VR-guided meditation.

#### Pain and EEG Signals

In terms of the secondary focus of the study to explore pain changes correlated with EEG variations, we found no significant association between pain reduction and changes in electrophysiological signal. This could be due to the limited sample size of this exploratory study. More electrophysiological studies on a larger sample population could potentially identify EEG correlates associated with pain reduction after VR-guided meditation.

### Limitations

As an exploratory study, the sample numbers were small and not necessarily representative of the wider population of patients with CP, which limited the power to identify differences. Therefore, there is a need for neurophysiological studies with larger samples to validate these results and to better explore this phenomenon. In addition, this was a single cohort study with no comparison group, although resting states before and after meditation were used as a no-mindfulness within-subjects control. Finally, the study focused on short-term neurophysiological alterations in the electrophysiology of the brain, and the long-term effects of meditative VR environments are still unknown, which will require longitudinal studies.

### Conclusions

These findings suggest that distinct altered neurophysiological brain signals are detectable during VR-guided meditation, predominantly in terms of an increase in the power of the β and γ bands. Changes in the α and θ bands were also identified, predominantly as a pattern in VR-guided meditation compared with the resting baseline, possibly reflecting the specific impact of visual activity during VR-guided meditation. Some changes in coherence were also observed between the frontal and parietal and occipital cortices during VR-guided meditation. No significant association between NRS pain scores and changes in EEG signals was observed. Although this is an exploratory study, the results of this work clearly demonstrate the feasibility of EEG recording and subsequent data processing and analysis during VR experiences in patients using modern VR HMDs. To our knowledge, this is the first exploration of EEG alterations in the brain’s electrophysiological signals associated with VR-guided meditation in patients with CP and should provide some valuable initial data to inform future work in this field.

## Abbreviations

CP: chronic pain
CMS: Command Mode Sense
DRL: Driven Right Leg
EEG: electroencephalograph
FDR: false discovery rate
HMD: head-mounted display
ICA: independent component analysis
LC: left central
LF: left frontal
LP: left parietal
MBSR: mindfulness-based stress reduction
MANOVA: multivariate analysis of variance
NRS: numerical rating scale
PSD: power spectral density
RCT: randomized controlled trial
RF: right frontal
VR: virtual reality
